# Phytochemical Constituents, Antioxidant, Cytotoxic, and Antimicrobial Activities of the Ethanolic Extract of Mexican Brown Propolis

**DOI:** 10.3390/antiox9010070

**Published:** 2020-01-13

**Authors:** J. Fausto Rivero-Cruz, Jessica Granados-Pineda, José Pedraza-Chaverri, Jazmin Marlen Pérez-Rojas, Ajit Kumar-Passari, Gloria Diaz-Ruiz, Blanca Estela Rivero-Cruz

**Affiliations:** 1 Facultad de Química, Universidad Nacional Autónoma de México, Ciudad Universitaria, Ciudad de México 04510, Mexico; jessygpin@hotmail.com (J.G.-P.); pedraza@unam.mx (J.P.-C.); gloriadr@unam.mx (G.D.-R.); blancariv@unam.mx (B.E.R.-C.); 2 División de Investigación Básica, Instituto Nacional de Cancerología, Av. San Fernando 22, Apartado Postal 22026, Tlalpan 14000, Mexico; jazminmarlen@gmail.com; 3 Instituto de Investigaciones Biomédicas, Universidad Nacional Autónoma de México, Ciudad Universitaria, Ciudad de México 04510, Mexico; ajit.passari22@iibiomedicas.unam.mx

**Keywords:** propolis, antioxidant activity, cytotoxic, antibacterial, México, HS-SPME/GC-MS-TOF, NMR, volatile compounds, flavonoids, phenolic acids

## Abstract

Propolis is a complex mixture of natural sticky and resinous components produced by honeybees from living plant exudates. Globally, research has been dedicated to studying the biological properties and chemical composition of propolis from various geographical and climatic regions. However, the chemical data and biological properties of Mexican brown propolis are scant. The antioxidant activity of the ethanolic extract of propolis (EEP) sample collected in México and the isolated compounds is described. Cytotoxic activity was evaluated in a central nervous system and cervical cancer cell lines. Cytotoxicity of EEP was evaluated in a C6 cell line and cervical cancer (HeLa, SiHa, and CasKi) measured by the 3-(3,5-dimethylthiazol-2-yl)2,5-diphenyltetrazolium (MTT) assay. The antibacterial activity was tested using the minimum inhibitory concentration (MIC) assay. Twelve known compounds were isolated and identified by nuclear magnetic resonance spectroscopy (NMR). Additionally, forty volatile compounds were identified by means of headspace-solid phase microextraction with gas chromatography and mass spectrometry time of flight analysis (HS-SPME/GC-MS-TOF). The main volatile compounds detected include nonanal (18.82%), α-pinene (12.45%), neryl alcohol (10.13%), and α-pinene (8.04%). EEP showed an anti-proliferative effect on glioma cells better than temozolomide, also decreased proliferation and viability in cervical cancer cells, but its effectiveness was lower compared to cisplatin.

## 1. Introduction

Propolis also known as “bee glue” is a nontoxic hive product accumulated by bees from diverse plants containing compounds such as flavonoid aglycones, phenolic acids and their esters, phenolic aldehydes, alcohols, ketones, sesquiterpenes, coumarins, steroids, amino acids, and inorganic compounds. It functions in sealing holes, cracks, reconstruction, and smothering the inner surfaces of the beehive. Propolis and its extracts have application in treating diseases due its anti-inflammatory, antioxidant, antibacterial, antimycotic, antifungal, antiulcer, anticancer, and immunomodulatory properties [1,2]. Egyptians, Greeks, Romans, Chinese, Arabs, and Incas have traditionally used it as an antiseptic to treat wounds and as an anti-pyretic agent [1,2,3]. The term propolis was described in the 16th Century in France and was considered as an official drug by London Pharmacopoeia [4].

Propolis contains mainly hydrophobic terpenes and phenolics compounds and can be classified in five types. The chemogeographic patterns of propolis types reflects the geographical distribution of plants species [2]. According to this distribution, the propolis produced in North America belongs to the poplar propolis and contains mainly flavonoids without B-ring substituents, such as pinocembrin, pinobanksin, galangin, and chrysin [2,5].

In recent years, it has gained wide acceptance as traditional medicine in various parts of the world where it is claimed to improve human health and to prevent diseases such as diabetes and cancer [6,7]. Nowadays, in several countries, it is possible to find propolis as liquid extracts in bottles, vaporizers, syrups capsules, tablets, candies, creams, among others [8,9].

Data about the chemical composition and biological activity of propolis from México is limited [10,11,12]. Propolis in Atliplano region is prepared in several forms, including syrups, tinctures, and creams as an alternative to improve health and prevent diseases. In view of the importance of propolis in Mexican traditional medicine the chemical composition and biological activities (antioxidant, antibacterial, and cytotoxic) of the ethanolic extract of Altiplano propolis (EEP) were investigated.

The aims of this study were: (a) To isolate the major compounds of brown Mexican propolis, and (b) to evaluate the antibacterial, antioxidant, and cytotoxic activities of the EEP.

## 2. Materials and Methods

### 2.1. Chemicals and Reagents

All the chemicals to investigate the antioxidant, antibacterial, and cytotoxic activities were supplied by Sigma-Aldrich (St. Louis, MO, USA). All the solvents and chromatographic supports were purchased from Merck (Darmstadt, Germany). Deuterated solvents and TMS were provided by Cambridge Isotope Laboratories (Tewksbury, MA, USA).

### 2.2. Instrumentation

Nuclear magnetic resonance (NMR) spectra were taken on a Bruker AVANCE III 400 (400 MHz) spectrometer, using tetramethylsilane (TMS) as an internal standard.

### 2.3. Propolis Samples 

Beekeepers kindly provided four raw propolis samples from Silao and Irapuato, Guanajuato, México (Table 1). The samples were harvested using traps in 2018 and 2019 and were frozen and stored at −20 °C until analysis.

### 2.4. Antioxidant Activity

The EEP antioxidant activity was assessed using two different assays in vitro: DPPH and ABTS. Both methods were modified and translated into 96-well plates. Each test was done in three replicates.

Radical scavenging activity (RSA) for DPPH was evaluated according to the method described in [11]. Briefly, an ethanolic solution of 0.208 mM was added to 0.1 mL of different concentrations of extracts and pure compounds. The 96-well plate was maintained in a dark at room temperature for 20 min and the absorbance was recorded at 540 nm. The RSA was calculated as: RSA = 100 × (A_control_ − A_sample_)/A_control_, where A_control_ and A_sample_ are the absorbance. The IC_50_ values were calculated from the relationship curve of RSA versus concentrations of the respective sample curve. 

The ABTS test was performed according to the methodology previously reported in [13,14] and slightly modified. The RSA of the ABTS radical was calculated using the following equation: % inhibition = 100 (A_control_ − A_sample_)/A_control_. The IC_50_ was calculated from the scavenging activities (%) versus concentrations of the respective sample curve.

### 2.5. Total Phenol and Total Flavonoid Content

In this paper, we used spectrometric procedures for the quantification of the total phenolic and flavonoids content in propolis. The total phenol content in extracts was adapted from the method described by Singleton and Rossi [15]. The total flavonoid content was determined using the aluminum chloride reagent and the method described by Marquele et al. [16]. The total phenol content was expressed as mg equivalents of gallic acid/g of dry extract of propolis (EEP). The total flavonoid content was expressed as mg equivalents of quercetin/g of dry extract of propolis (EEP).

### 2.6. Extraction and Isolation of Compounds 1–12 from EEP GUA-4

The air-dried and powdered propolis samples GUA-4 were extracted with ethanol for up to one week and the resultant extract was concentrated in vacuo. A portion of ethanol-soluble extract (10.3 g) was subjected to a silica gel vacuum column chromatography (VLC) and eluted with a gradient mixture of dichloromethane–acetone (1:0 → 0:1) to give eight pooled fractions (F2–F8). Fractions F2, F4, and F7 showed the best antioxidant activity. Fraction F2 was chromatographed over a Sephadex LH-20 column and eluted with methanol to yield **1** (40 mg). Fraction F4, eluted with dichloromethane-acetone (9:1), was chromatographed over a Sephadex LH-20 column, using methanol as eluent, to give six fractions. Fraction F4-3 (100 mg) was separated by TLC with dichloromethane–acetone (99:1), followed by TLC with dichloromethane–acetone (98:2), to give **2** (20 mg) and **3** (65 mg). Fraction F5 was rechromatographed on a Sephadex LH-20 column using methanol as solvent to give six subfractions (F5-1: 10 mg; F5-2: 12.3 mg; F5-3: 17.5 mg; F5-4: 15.7 mg; F5-5: 25.3 mg; F5-6: 18.1 mg). Subfraction F5-1 yielded crystals of 4. F5-5 (25.3 mg) yielded crystals of 5 (9.0 mg). Mother liquor was subjected to a preparative thin-layer chromatography (PTLC) with acetone-toluene (5:95) to give 6. Subfraction F5-6 (18.1 mg) yielded crystals of 7 (11.0 mg). Fraction F7 was chromatographed on silica gel using a hexane–EtOAc gradient system to give five fractions (F7-1: 13.9 mg; F7-2: 517.7 mg; F7-3: 28.4 mg; F7-4: 16.7 mg; F7-5: 32.8 mg; F7-6: 56.8 mg; F7-7: 45.1 mg). Subfraction F7-1 was identified as 8. Subfraction F7-2 was chromatographed by RP-MPLC, using H_2_O–CH_3_CN (1:0 → 0:1) to afford five subfractions (F7-2.1: 125.3 mg; F7-2.2: 275.1 mg; F7-2.3: 67.8 mg; F7-2.4: 37.0 mg; F7-2.5: 12.3 mg). Subfraction F7-2.3 was identified as 9. Subfractions F7-2.4, F7-2.5, and F7-2.6 were dissolved in CH_2_Cl_2_–MeOH and left overnight to give crystals of 10, 11, and 12, respectively.

### 2.7. Head Space Solid Phase Microextraction (HS-SPME), GC-MS Analysis, and Identification of Volatile Components

The HS-SPME was performed using a Stableflex^®^ fiber 50/30 μm DVB/CAR/PDMS (1 cm) as described in our previous work [17].

The analysis of volatile compounds was carried out on a GC Agilent 6890 N (Agilent Technology, Santa Clara, CA, USA) series gas chromatograph coupled to a LECO time of flight mass spectrometer (LECO Corporation, St. Joseph, MI, USA). The volatile compounds were separated on a 5% diphenyl-95% dimethyl polysiloxane (30 m × 0.18 mm i.d.; 0.18 μm film thickness) capillary column (Bellefonte, PA, USA). The carrier gas was helium with a flow rate of 1 mL/min and split ratio of 1:50. The column initial temperature was 40 °C. It was then raised to 300 °C with a rate of 20 °C/min and was held for 5 min. The ionization electron energy was 70 eV and the mass range scanned was 40–400 *m*/*z*. The injector and MS transfer were set at 300 and 250 °C, respectively. The volatile constituents of propolis were identified by co-injection of the sample with standard samples when available; based on their Kovats Index, calculated in relation to the retention times of a series of alkanes (C-8–C-20), in comparison with those of the chemical compounds gathered by Adams [18] and by comparing their MS fragmentation patterns with those of pure compounds in the spectral database of the National Institute of Standards and Technology (NIST) [19].

### 2.8. Cell Culture

Rat C6 glioma cell and human cervical cancer cell lines (HeLa, SiHa, and CaSki) were obtained from American Type Culture Collection (Manassas, VA, USA). Cells were routinely maintained in Dulbecco’s modified Eagle´s medium (DMEM) supplemented with fetal bovine serum (Gibco BRL) with 5% for C6 cells and 10% for cervical cancer cells, and incubated at 37 °C in an atmosphere comprising 5% CO_2_ and 95% air at high humidity. Cells were harvested with 0.025% trypsin and 0.01% EDTA (Gibco BRL).

The effect of EEP, cisplatin, and temozolomide on the proliferation of cells were evaluated using the MTT assay (3-(3,5-dimethylthiazol-2-yl)2,5-diphenyltetrazolium), which is based on the reduction of a tetrazolium salt in metabolically active cells. The procedure was as follows. Viable cells were seed into 96-well plates in 100 µL per well of DMEM culture medium at a density of 3 × 10^3^ for C6 cells, 2 × 10^4^ for CaSki cells, and 1 × 10^4^ cells for HeLa and SiHa cells. After treatment, the medium was removed and the MTT solution was added to each well, followed by 1 to 2 h in a humidified atmosphere containing 5% CO_2_ at 37 °C. The absorbance of the samples was measured spectrophotometrically at λ 570 nm using a microtiter plate ELISA reader. Results are expressed as the percentage of MMT reduction. 

C6 cells were exposed for 72 h with 2 to 200 µg/mL of EEP, since it is the time used to make the exposure with temozolomide (first line treatment used for glioblastoma), whereas HeLa, SiHa, and CaSki cells were exposed for 24 h with 15 to 500 µg/mL of EEP; after that time, cell viability was quantified using the MTT assay. Temozolomide (250 µM) and cisplatin (5–320 µM) were used as a control. The concentration of drugs to reach 50% growth inhibition (IC_50_) was obtained from the survival curves. The experiments were conducted in triplicate in independent experiments. Values are expressed as the mean ± SEM of at least three independent experiments. SigmaPlot 12.3 software (Systat Software, Santa Clara, CA, USA) was used.

### 2.9. Antibacterial Activity

The in vitro antibacterial activity of EEP and compounds **1**–**4**, **10**–**13**, and **15**–**17** were determined using a broth microdilution test as recommended by Clinical and Laboratory Standards Institute (New York, NY, USA) M7-A11 for bacteria [20]. The minimum inhibitory concentration (MIC) was defined as the lowest concentration of the test agent that had restricted growth to a level <0.05 at 660 nm after incubation at 37 °C for 16–24 h.

## 3. Results and Discussion

### 3.1. Total Phenol and Flavonoid Content

The total polyphenol contents for the selected propolis samples were found to be 178.9 ± 5.7, 198.4 ± 4.1, 167.6 ± 7.8, and 246.3 ± 3.2 mg GAE/g of dry extract for GUA-1, GUA-2, GUA-3, and GUA-4, respectively. It should be noted that the Folin-Ciolcateau reagent was reported in the literature as not specific to only phenols and could react with other reducing compounds that could be oxidized by the Folin reagent [21]. The total flavonoid contents were 64.32 ± 5.75, 58.34 ± 2.8, 77.45 ± 6.9, and 87.5 ± 1.9 mg QE/g of dry extract for GUA-1, GUA-2, GUA-3, and GUA-4, respectively. Flavonoids can form complexes with aluminum chloride to yield a yellow solution. Valencia et al. [22] has been reported values of 629.6 ± 9.9 mg GAE/g of dry extract for TPC and 185.9 ± 3.2 mg QE/g of dry extract and TF in a propolis from Sonora, México. The most important flavonoids isolated in this propolis sample were pinocembrin, pinobanksin 3-acetate, and chrysin. The results also confirm the influence of the geographic region and the season of collection for the quality and properties of the propolis [2].

### 3.2. Antioxidant Activity Assays

The EEP was evaluated for its ability to quench the DPPH^.^, which is one of the few stable organic nitrogen radicals and bears a purple colour. This assay is based on the measurement of the loss of DPPH^•^ after reaction with samples. It is considered as the prior mechanism involved in the electron transfer. The IC_50_ value is a parameter widely used to measure the antioxidant activity of test samples. It is calculated as the concentration of antioxidants needed to decrease the initial DPPH concentration by 50% [23]. Thus, the lower IC_50_ value the higher antioxidant activity. The EEP GUA-4 showed an antioxidant activity (IC_50_ = 67.9 μg/mL) comparable to the reference ascorbic acid (IC_50_ = 43.2 μg/mL) and tenfold lower than Trolox (IC_50_ = 6.3 μg/mL) and seven-fold lower than quercetin (IC_50_ = 9.9 μg/mL). While caffeic acid (12) showed the highest activity (IC_50_ = 5.9 μg/mL) along with ferulic acid (10) (IC_50_ = 9.9 μg/mL) and syringic acid (11) (IC_50_ = 9.8 μg/mL). The lowest antioxidant activity corresponds to 5-methylchrysin ether (9) (IC_50_ = 112.9 μg/mL) and 5-methyl-pinobanksin ether (5) (IC_50_ = 98.4 μg/mL).

There are reports of the DPPH scavenging capacity, in terms of IC_50_, for pinocembrin (**1**), chrysin (**2**), galangin (**3**), isorhamnetin (**7**), ferulic acid (**10**), syringic acid, (**11**) and caffeic acid (**12**) [23,24,25,26]. For alpinetin (**4**) only the percentage of scavenging activity of DPPH is reported [27]. Nevertheless, the information available for dillenetin (**6**), 5-methyl-pinobanksin ether (**5**), 5-methylchrysin ether (**9**), and 5-methylgalangin ether (**8**) is scarce. Thus, the present work stands for the first report of the IC_50_ as a measurement of their antioxidant activity (Table 2). 

In the ABTS assay, the antioxidant activity is measured as the ability of test compounds to decrease the color reacting directly with the radical ABTS•^+^ [28]. Ferulic acid (**10**), syringic acid (**11**), caffeic acid (**12**), and chrysin (**2**) showed the lowest IC_50_ (Table 2). The EEP, 5-methylchrysin ether (**9**), and 5-methyl-pinobanksin ether (**5**) showed weak antioxidant activity (Table 2).

The analysis of their chemical structures can explain the weak antioxidant activity of the methylated flavonoids. According to Procházková et al. [29] the catechol structure in the B-ring, 2,3-double bond in conjugation with a 4-oxo function in the C-ring and the hydroxyl groups in meta position in ring-A. On the other hand, the activity of phenolic acids lies on the presence of two *o*-hydroxyl groups in the aromatic ring.

### 3.3. Cytotoxicity of EEP on Cancer Cells

Cytotoxicity was expressed as the percentage grow inhibition of C6, HeLa, SiHa, and CaSki cells treated with EEP. In all the cases, EEP shows a cytotoxicity concentration-depended manner. In Table 3, we show the IC_50_ value of EEP over four cancer cell lines. Those results show that EEP restricts glioblastoma cells (C6 cell cancer line) proliferation in vitro as efficiently as temozolomide (reference drug), whereas, for cervical cancer cell lines, it requires a higher concentration of the EEP compared to cisplatin.

There are a few studies of beneficial properties of Mexican propolis. Li et al. [11] reported that three of the 39 compounds isolated from the methanolic extract of Mexican propolis exhibited a potent cytotoxic effect in a colon, melanoma, lung, cervix, and fibrosarcoma cancer cell lines. Li et al. reported the isolation of flavonoids from methanolic extract of Mexican propolis, and one of them revealed significant cytotoxic effect against pancreatic human cancer cell line with IC_50_ values of 4 μM [10]. Other studies described the potent cytotoxic activity of galangin (**3**); ferulic acid (**10**); syringic acid (**11**); and caffeic acid (**12**) against different cancer cell lines [22]. Interestingly, a few researchers reported that these compounds could be useful for therapeutic treatments. For example, Benguedouar et al. [30] reported that ethanolic extract of Algerian propolis (EEP) and galangin (**3**) decreased the number of B16F1 melanoma cells in vitro compared to control. Celinska-Janowicz et al. [31] state that the ethanolic extract of propolis isolated abundant polyphenolic compounds such as ferulic acid (**10**) and caffeic acid (**12**) revealed pro-apoptotic activity on human tongue squamous carcinoma cells (CAL-27). From this information, we emphasized that EEP and compounds possesses anti-cancer effects against cancer cell lines.

### 3.4. Antibacterial Activity

As shown in Table 4, antimicrobial screening against four oral pathogens revealed that compounds **1**, **3**, **4**, **12**, and **16** were inhibitory to the growth of *Streptococcus mutans*, *Streptococcus oralis*, *Streptococcus sanguinis,* and *Phorphyromonas gingivalis*. Among these, compounds **1**, **3**, **4**, and **12**, were either equally or more potent than their respective crude extract of origin (Table 4). Earlier in vitro studies have shown that the Sonoran ethanolic extract of propolis exhibited antibacterial activity against *E. coli* (ATCC 25922) and *S. aureus* (ATCC 6538P). The propolis constituents CAPE, pinocembrin, pinobanksin 3-*O*-acetate, and naringenin exhibited significant inhibitory activity on the growth of *S. aureus*. CAPE exhibited the maximum inhibitory effect on the bacterial growth (CAPE (MIC 0.1 mmol/L), pinocembrin (MIC 0.4 mmol/L), pinobanksin 3-*O*-acetate (MIC 0.8 mmol/mL), and naringenin (0.8 mmol/L)). None of the propolis constituents influenced the growth of *E. coli* at any of the tested concentrations [32].

### 3.5. Chemical Composition of EEP GUA-4

The EtOH-soluble extract of sample GUA-4 was fractionated by chromatography on a VLC column and by Sephadex LH-20, giving 12 known compounds (**1**–**12**). The compounds were identified as pinocembrin (**1**, 405.4 mg) [33], chrysin (**2**, 126.3 mg) [34], galangin (**3**, 56.7 mg) [33], alpinetin (**4**, 15.6 mg) [35], 5-methyl-pinobanksin ether (**5**, 16.8 mg) [36], dillenetin (**6**, 12.3 mg) [37], isorhamnetin (**7**, 24.6 mg) [38], 5-methylgalangin ether (**8**, 9.8 mg) [39], 5-methylchrysin ether (**9**, 16.3 mg) [40], ferulic acid (**10**, 24.9 mg) [33], syringic acid (**11**, 6.7 mg), and caffeic acid (**12**, 8.9 mg) [33] (Figure S1) by means of 1D (Table S1) and 2D NMR spectral analysis. The presence of pinocembrin (**1**), chrysin (**2**), galangin (**3**), ferulic acid (**10**), syringic acid (**11**), and caffeic acid (**12**) suggest that its main botanical source are a species of *Populus* typical of the country, such as *Populus mexicana* Wesmael, *Populus guzmanantlensis* Vazques and Cuevas and *Populus simaroa* Rzedowski. Although flavonoids without B-ring substituents appear common in temperate propolis from both the northern and southern hemispheres, in this study we report for the first time the presence of dillenetin (**6**) and isorhamnetin (**7**) with B-ring substitution as constituents of Mexican poplar propolis. To the best of our knowledge, this is the first report of dillenetin (**6**) as a propolis constituent. 5-methylpinobanksin (**5**), alpinetin (**4**), isorhamnetin (**7**), 5-methylgalangin ether (**8**), and 5-methylchrysin ether (**9**) were previously identified by diode array detection and ESI mass spectrometry (LC-DAD-ESI-MS) as constituents of Italian, Portuguese, and Czech propolis [41,42]. 

### 3.6. Volatile Compounds

Forty volatile constituents were identified as shown in Table 5. The typical analytical ion current (AIC) chromatogram is shown in Figure S2. The chemical composition of volatiles was found in agreement with previous reports [43]. The main volatile compounds identified were nonanal (18.829%, **13**), β-pinene (12.179%, **14**), 1-octen-3-ol (12.129%, **15**), neryl alcohol (10.135%, **16**), α-pinene (8.046%, **17**), 6-methyl-3,5-heptadiene-2-one (6.803%, **18**), *p*-cymen-9-ol (3.108%, **19**), and sylvestrene (3.022 %, **20**) (Figure S3). It is important to note that the content of the compounds differed from a previous study carried out on the volatile compounds in propolis samples from Yucatan (México). In that sample, hexadecanoic acid (10.9%) and trans-verbenol (7.0%) were the predominant compounds [44].

## 4. Conclusions

In the present study, we have isolated twelve (**1**–**12**) components and identified 40 volatile compounds in Mexican propolis. The current study revealed the presence of antioxidant, antimicrobial, and cytotoxic phytochemicals [galangin (**3**); ferulic acid (**10**); syringic acid (**11**); and caffeic acid (**12**)] in EEP. It is concluded that EEP can be a potential addition in pharmaceutical products for the improvement of human health by contributing in the antioxidant defense system fighting against the production of free radicals. México, being a megadiverse country, has numerous numbers of propolis differing in chemical composition. However, unfortunately it is still unexplored.

## Figures and Tables

**Table 1:** Location of recollection, harvesting method, and weight recollected.

Code	Location of Recollection	Year of Recollection	Harvesting Method	Weight (g)
GUA-1	Silao	2018	plastic nest	6.0
GUA-2	Irapuato	2018	plastic nest	8.50
GUA-3	Irapuato	2019	plastic nest	6.95
GUA-4	Silao	2019	plastic nest	32.0

**Table 2:** Scavenging ability of ethanol extract of propolis (EEP) GUA-4 and compounds **1**–**12**.

Compounds	IC_50_ (μg/mL)	
	DPPH	ABTS
EEP GUA-4	67.9 ± 0.1	98.7 ± 0.5
pinocembrin (**1**)	23.5 ± 1.8	44.8 ± 2.1
chrysin (**2**)	10.7 ± 0.2	16.3 ± 0.8
galangin (**3**)	15.3 ± 0.5	26.8 ± 0.1
alpinetin (**4**)	47.3 ± 1.9	69.5 ± 0.7
5-methylpinobanksin ether (**5**)	98.4 ± 2.3	126.9 ± 4.5
dillenetin (**6**)	35.7 ± 3.5	48.9 ± 2.9
isorhamnetin (**7**)	21.4 ± 3.1	36.7 ± 4.6
5-methylgalangin ether (**8**)	63.2 ± 2.6	94.2 ± 6.2
5-methylchrysin ether (**9**)	112.9 ± 1.9	158.4 ± 4.9
ferulic acid (**10**)	9.9 ± 0.7	16.7 ± 0.2
syringic acid (**11**)	9.8 ± 0.4	13.0 ± 0.9
caffeic acid (**12**)	5.9 ± 0.4	9.7 ± 0.5
Quercetin	9.9 ± 2.5	16.1 ± 2.1
Trolox	6.3 ± 1.4	3.8 ± 1.2
ascorbic acid	43.2 ± 10.3	36.8 ± 2.5

**Table 3:** Cytotoxic effect of EEP on several cancer cell lines.

Cell Line	IC_50_	Reference Drug	Reference Concentration
C6	92.2 µg/mL	Temozolamide	IC_30_ 250 µM (50 µg/mL)
HeLa	>100 µg/mL (357 µg/mL)	Cisplatin	IC_50_ 46 µM (14 µg/mL)
SiHa	>100 µg/mL (500 µg/mL)		IC_50_ 121 µM (36 µg/mL)
CaSki	>100 µg/mL (538 µg/mL)		IC_50_ 163 µM (50 µg/mL)

**Table 4:** Antimicrobial activity of ethanolic extract and compounds **1**–**4**, **10**–**13**, **15**–**17**, and chlorhexidine digluconate (CHX) ^a^.

Compounds	MIC ^b^ (μg/mL)			
	*S. mutans*	*S. oralis*	*S. sanguinis*	*P. gingivalis*
EEP	250	125	125	500
EOP	500	500	500	1000
pinocembrin (**1**)	256	128	128	512
chrysin (**2**)	512	512	512	1024
galangin (**3**)	256	256	256	1024
alpinetin (**4**)	128	256	128	516
ferulic acid (**10**)	500	250	250	500
syringic acid (**11**)	250	250	250	500
caffeic acid (**12**)	128	128	128	256
nonanal (**13**)	256	256	128	512
neryl alcohol (**15**)	1024	512	512	1024
α-pinene (**16**)	250	250	250	500
α-pinene (**17**)	500	500	500	500
^a^ CHX	0.02	0.02	0.02	0.12

^a^ positive control; ^b^ minimum inhibitory concentration.

**Table 5:** Volatile components from Mexican propolis GUA-4.

	Name	Retention Index	Area %	Method of Identification
1	α-pinene	939	8.046	a, b, c
2	β-pinene	979	12.179	a, b, c
3	1-octen-3-ol	982	12.129	a, b
4	ethyl hexanoate	1003	0.042	a, b, c
5	Octanal	1006	1.273	a, b
6	Sylvestrene	1030	3.022	a, b
7	acetophenone	1076	0.486	a, b
8	1-octanol	1078	0.107	a, b
9	Thujone	1102	0.276	a, b
10	*p*-cymenene	1082	0.291	a, b
11	Nonanal	1100	18.829	a, b, c
12	6-methyl-3,5-heptadiene-2-one	1105	6.803	a, b
13	Eucalyptol	1039	0.472	a, b, c
14	Camphor	1146	0.583	a, b, c
15	*trans*-pinocamphone	1162	2.386	a, b
16	*trans*-terpineol	1163	0.102	a, b
17	*p*-menth-1,5-dien-8-ol	1167	1.097	a, b
18	(2*E*)-nonenal	1168	1.097	a, b
19	*m*-cymen-8-ol	1180	1.024	a, b, c
20	Unknown 1	1185	0.064	-
21	α-terpineol	1189	1.571	a, b
22	Myrtenol	1193	1.571	a, b
23	d-fenchone	1187	1.828	a, b
24	*p*-cymen-9-ol	1207	3.108	a, b, c
25	neryl alcohol	1217	10.135	a, b
26	trans-carveol	1219	2.953	a, b
27	Citronellol	1228	1.916	a, b
28	Ocimenone	1230	0.727	a, b
29	*cis*-chrysanthenyl acetate	1253	0.243	a, b
30	Geraniol	1278	0.314	a, b
31	neodehydro carveol acetate	1307	1.843	a, b
32	ethyl nonanoate	1319	1.283	a, b
33	trans-carvyl acetate	1337	0.357	a, b
34	α-longipinene	1352	0.181	a, b
35	β-cububene	1388	0.248	a, b
36	β-bourbonene	1398	0.217	a, b
37	n-decyl acetate	1408	0.109	a, b
38	trans-geranylacetone	1455	0.429	a, b
39	γ-gelinene	1485	0.103	a, b
40	β-bisabolene	1509	0.290	a, b

a: Retention time; b: Retention index; c: Mass spectrum.

## Supplementary Materials

The following are available online at https://www.mdpi.com/2076-3921/9/1/70/s1, Figure S1: Flavonoids isolated from EEP GUA-4; Figure S2: Analytical ion current chromatogram (AIC) of propolis GUA-4; Figure S3: Major volatile compounds of EEP GUA-4; Table S1: ^1^H-RMN data of the flavonoids isolated from Mexican propolis.
