# Supplementary material for: Phytochemical Constituents, Antioxidant, Cytotoxic, and Antimicrobial Activities of the Ethanolic Extract of Mexican Brown Propolis
Source: Antioxidants (Basel). 2020 Jan 13;9(1):70. doi: 10.3390/antiox9010070 (PMC7022611; doi:10.3390/antiox9010070)
Supplement: Supplementary file 1 [file antioxidants-09-00070-s001.zip › antioxidants-669539-supplementary.pdf]

Phytochemical constituents, antioxidant, cytotoxic and antimicrob[illegible] of the ethanolic extract of Mexican brown propolis.

J. Fausto Rivero-Cruz[1,*], Jessica Granados-Pineda[1], José Pedraza-Chaverri[1], Jazmin M. Pérez-Rojas[2], Ajit Kumar-Pass[illegible] Blanca Estela Rivero-Cruz[1]

[1]*Facultad de Química, Universidad Nacional Autónoma de México, Ciudad Universitaria, 04510, Ciudad de México, México*; joserc@unam.mx (J.[illegible] jessygpin@hotmail.com (J. G-P.); pedraza@unam.mx (J. P-C.)(blancariv@unam.mx (B.E R-C.)

[2]*División de Investigación Básica, Instituto Nacional de Cancerología, Av. San Fernando 22, Apartado Postal 22026, Tlalpan 14000 Mexico City, M[illegible]*

[3]*Instituto de Investigaciones Biomédicas, Universidad Nacional Autónoma de México, Ciudad Universitaria, 04510, Ciudad de México, México.* ajit.passari22@iibiomedicas.unam.mx

*Correspondence: joserc@unam.mx; Tel.: +52-55-5622-5281 (J.F.R-C.)

Received: date; Accepted: date; Published: date

Abstract: Propolis is a complex mixture of natural sticky and resinous components produced by honeybees from living [illegible] research has been dedicated to studying the biological properties and chemical composition of propolis from various g[illegible] regions. However, the chemical data and biological properties of Mexican brown propolis are scant. The antioxidant activi[illegible] of propolis (EEP) sample collected in Mexico and the isolated compounds is described. Cytotoxic activity was evaluated in [illegible] and cervical cancer cell lines. Cytotoxicity of EEP was evaluated in C6 cell line and cervical cancer (HeLa, SiHa, and CasKi[illegible] dimethylthiazol-2-yl)2,5-diphenyltetrazolium (MTT) assay. The antibacterial activity was tested using the minimum inhibi[illegible] assay. Twelve known compounds were isolated and identified by nuclear magnetic resonance spectroscopy (NMR). Ac[illegible] compounds were identified by means of headspace-solid phase microextraction with gas chromatography and mass sp[illegible] analysis (HS-SPME/GC-MS-TOF). The main volatile compounds detected include nonanal (18.82%), α-pinene (12.45%), n[illegible] α-pinene (8.04%). EEP showed an anti-proliferative effect on glioma cells better than temozolomide, also decreased prol[illegible] cervical cancer cells, but its effectiveness was lower in compared to cisplatin.

Keywords: Propolis, antioxidant activity, cytotoxic, antibacterial, Mexico, HS-SPME/GC-MS-TOF, NMR, volatile compou[illegible] acids.

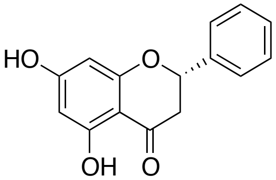

1

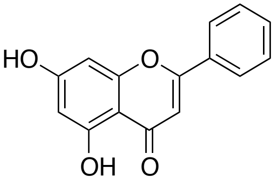

2

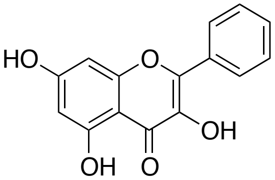

3

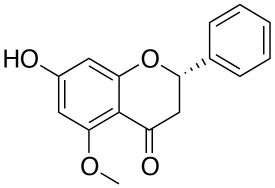

4

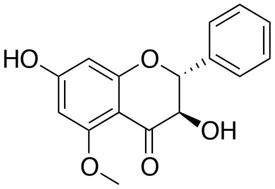

5

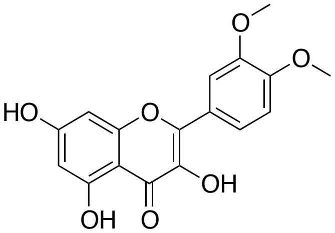

6

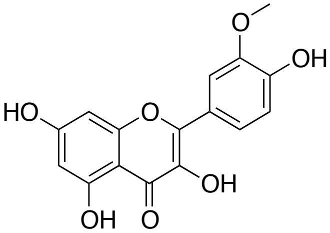

7

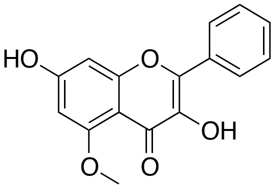

8

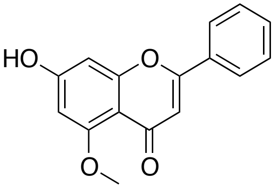

9

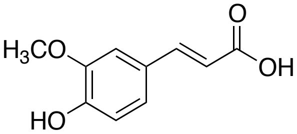

10

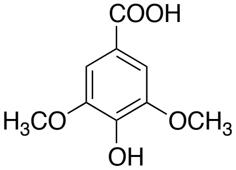

11

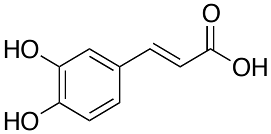

12

Figure S1. Flavonoids isolated from EEP GUA-4

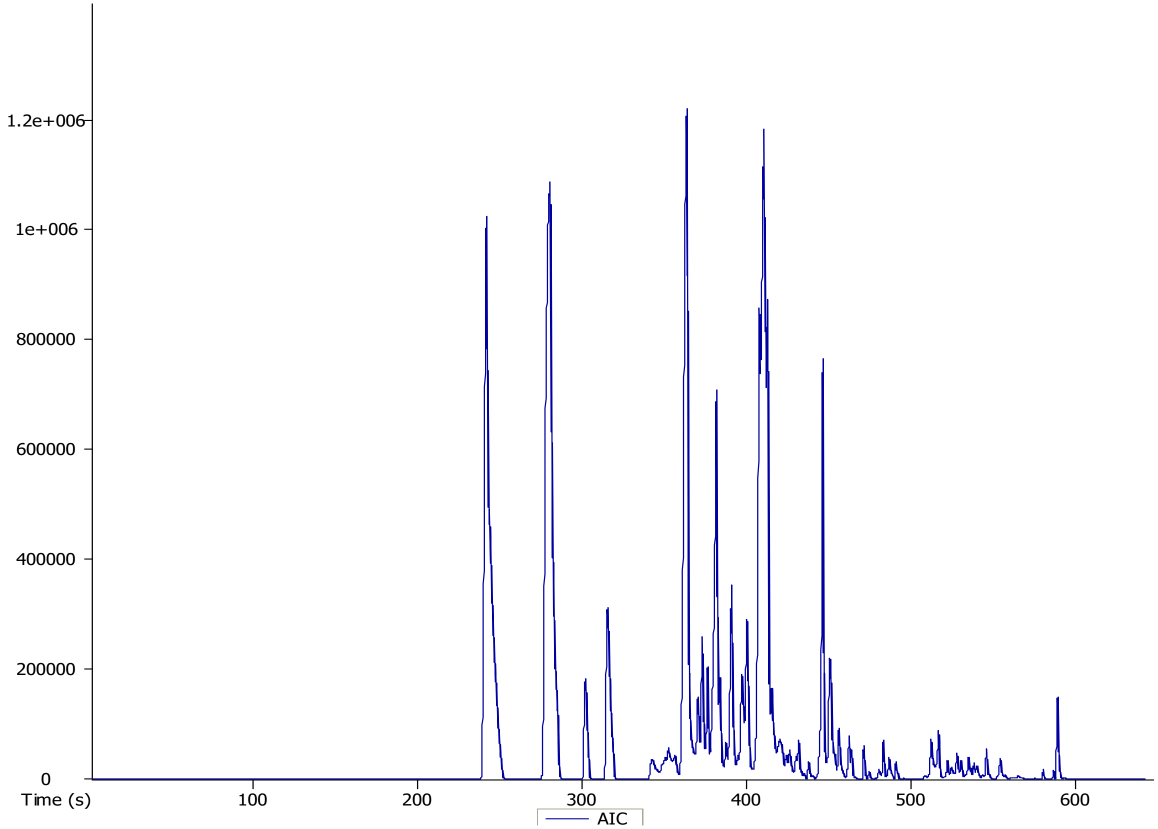

Figure S2. Analytical ion current chromatogram (AIC) of propolis GUA-4.

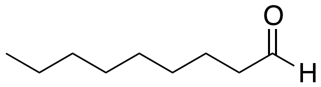

13

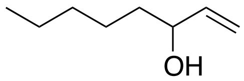

14

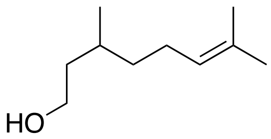

15

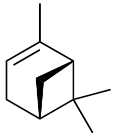

16

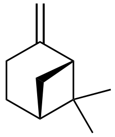

17

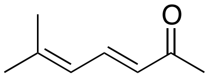

18

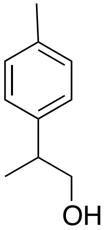

19

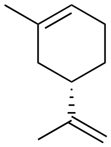

20

Figure 3. Major volatile compounds of EEP GUA-4.

Table S1. ^{1}H NMR (400 MHz) of the compounds isolated from propolis.

1H	pinocembrin (**1**) (MeOH-*d*$_4$)	chrysin (**2**) (DMSO-*d*$_6$)	alpinetin (**4**) (DMSO-*d*$_6$)	5-*O*-metilpinobanksin ether (**5**) (DMSO-*d*$_6$)
2	5.45 (1H, dd, *J*=16.0, 4.0)	-	5.46 (1H, dd, *J*= 16.0, 4.0 Hz)	4.32 (1H, d, *J*= 12.0 Hz)
3	3a 2.76 (1H, dd, *J*= 4.0, 16.0 Hz)<br>3b 3.09 (1H, dd, *J*= 16.0, 12.0 Hz)	6.98 (1H, s)	3a 2.60 (1H, dd, *J*= 4.0, 16.0 Hz)<br>3b 2.97 (1H, dd, *J*= 16.0, 12.0 Hz)	5.28 (1H, d, *J*= 12.0 Hz)
6	5.93 (1H, d, *J*= 4.0 Hz)	6.20 (1H, d, *J*= 2.0 Hz)	5.93 (1H, d, *J*= 2.0 Hz)	5.94 (1H, d, *J*= 2.0 Hz)
8	5.90 (1H, d, *J*= 4.0 Hz)	6.52 (1H, d, *J*= 2.0 Hz)	5.98 (1H, d, *J*= 2.0 Hz)	6.05 (1H, d, *J*= 2.0 Hz)
2´, 6´	7.49 (2H, dd, *J*= 8.0, 4.0 Hz)	8.1 (2H, dd, *J*= 8.0, 4.0 Hz)	7.48 (2H, dd, *J*= 8.0, 4.0 Hz)	7.48 (2H, dd, *J*= 8.0, 4.0 Hz)
3´-5´	7.39 (3H, m)	7.55 (3H, m)	7.41 (3H, m)	7.41 (3H, m)
5-OH	-	12.63 (1H, s)	-	-
5-OCH_3	-	-	3.72 (3H, s)	3.75 (3H, s)
3´-OCH_3	-	-	-	-
4´-OH	-	-	-	-

Table S1. ^{1}H NMR (400 MHz) of the compounds isolated from propolis (cont).

1H	dillenetin (**6**) (DMSO-*d*6)	Isorhamnetin (**7**) (DMSO-*d*6)	5-O-metil-galangin (8) (DMSO-*d*6)	5-O-methyl chrysin (9) (DMSO-*d*6)
2	-	-	-	-
3	-	-	-	6.68 (1H, s)
6	6.18 (1H, d, *J*= 2.0 Hz)	6.17 (1H, d, *J*= 2.0 Hz)	6.34 (1H, d, *J* = 2.0 Hz)	6.38 (1H, d, *J* = 4.0 Hz)
8	6.10 (1H, d, *J*=2.0 Hz)	6.45 (1H, d, *J*= 2.0 Hz)	6.47 (1H, d, *J* = 2.0 Hz)	6.55 (1H, d, *J* = 4.0 Hz)
2´	7.62 (1H, d, *J* =2.0 Hz)	7.76 (1H, d, *J*= 2.0 Hz)	8.11 (2H, d, *J* = 8.0 Hz)	7.98 (2H, m)
6´	7.56 (1H, dd, *J*= 8.0, 2.0 Hz)	7.67 (1H, dd, *J*= 8.0, 2.0 Hz)	8.11 (2H, d, *J* = 8.0 Hz)	7.98 (2H, m)
3´	-	-	7.43-7.54 (3 H, m)	7.43-7.54 (3H, m)
4´	-	-	7.43-7.54 (3H, m)	7.43-7.54 (3H, m)
5´	6.94 (1H, d, *J*= 8.0 Hz)	6.92 (1H, d, *J* = 8.0 Hz)	7.43-7.54 (3H, m)	7.43-7.54 (3H, m)
5-OH	12.65 (1H, s)	12.44 (1H, s)	-	-
5-OCH3	-	-	3.81 (3H, s)	3.78 (3H, s)
3´-OCH3	3.84 (3H,s)	3.82 (3H,s)	-	-
4´-OCH3	3.78 (3H,s)	-	-	-
